# Development and Validation of a Reversed-phase HPLC Method for Assay of the Decapeptide Cetrorelix Acetate in Bulk and Pharmaceutical Dosage Forms 

**Published:** 2014

**Authors:** Shirin Hooshfar, Seyed Alireza Mortazavi, Mohammad Piryaei, Hossein Ramandi Darzi, Nahid Shahsavari, Farzad Kobarfard

**Affiliations:** a *Department of Pharmaceutics, School of Pharmacy, Shahid Beheshti University of Medical Sciences, Tehran, Iran.*; b *Medical Genetics Department, Faculty of Medicine, Shahid Beheshti University of Medical Sciences, Tehran, Iran.*; c *Department of Clinical Pharmacy, School of Pharmacy, Shahid Beheshti University of Medical Sciences, Tehran, Iran.*; d *Department of Medicinal Chemistry, School of Pharmacy, Shahid Beheshti University of Medical Sciences, Tehran, Iran.*; e *Central Research Laboratories, Shahid Beheshti University of Medical Sciences, Tehran, Iran.*; f *Phytochemistry Research Center, Shahid Beheshti University of Medical Sciences, Tehran, Iran.*

**Keywords:** Cetrorelix acetate, HPLC, Assay, UV detection, Formulation

## Abstract

A gradient reversed-phase high performance liquid chromatography (HPLC) method was developed for the assay of cetrorelix acetate, a synthetic decapeptide with gonadotropin-releasing hormone (GnRH) antagonistic activity used in infertility treatment. The HPLC method, which is used to determine cetrorelix in bulk and pharmaceutical dosage forms, was validated per ICH guidelines. The chromatographic separation was achieved on a C18 reversed-phase column using acetonitrile, water and trifluoroacetic acid (TFA) as mobile phase and wavelength was set at 275 nm. The calibration curve was linear (r2 = 0.999) over cetrorelix concentrations ranging from 62.50 to 12.50 μg/mL (n = 6). The limits of detection (LOD) and quantification (LOQ) were calculated from the peak-to-noise ratio as 15.6 and 62.5 μg/mL, respectively. The method had an accuracy of > 97% and intra- and inter-day RSD of < 0.3% and < 1.6%, respectively and was validated with excellent specificity, sensitivity, and stability. The validated method was successfully applied for determination of cetrorelix in bulk and pharmaceutical dosage forms.

## Introduction

**Figure 1:**
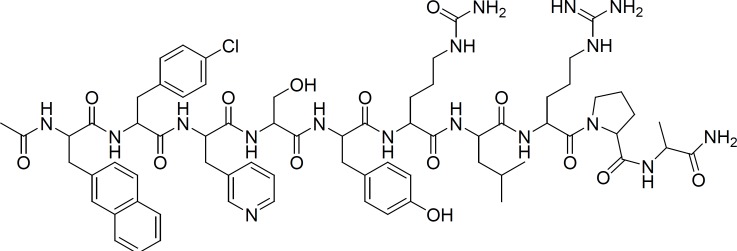
Chemical structure of cetrorelix

The decapeptide Cetrorelix ([Ac-D-Nal -(p-Cl)-D-Phe -D-Pal -Ser - Thy -D-Cit -Leu -Arg -Pro -D-Ala –NH2 ], Figure 1), is a third-generation Gn-RH antagonist that is currently mainly used in infertility treatment (for the prevention of premature ovulation in patients undergoing controlled ovarian stimulation) and is suitable for the treatment of benign prostatic hypertrophy and sexual hormone-dependent tumors (1, 2, 3, 4 and 5). 

Cetrorelix has been analyzed by HPLC using fluorescence, mass spectrometry, UV-detectoion and combinations of UV-detection and detection of radioactivity, for quantification in biological fluids, liposome and dry powder inhalation (1, 6, 7, 8, 9, 10 and 11). Fluorescence detection and mass spectrometry thechniquse suffer from not being commonly available in all analysis laboratories (12-13). Furthermore mass spectrometry method may have the highest sensitivity, but the determination process is complex (14-15). A HPLC method has been reported by Irngartinger *et al. *for determination of Cetrorelix in dry powder inhalation whit UV-detection but validation procedure has not been described (10). 

Unlike small organic molecules whose chromatographic behavior is described by a finite partitioning equilibrium between the stationary phase and the mobile phase, peptides typically do not exhibit such an effect. Instead, they exhibit an adsorption phenomenon in which the polypeptide is adsorbed onto the stationary phase and elutes only when the solvent strength of the mobile phase is sufficient to compete with the hydrophobic forces keeping it there (16-17). The sensitivity of peptide retention to subtle changes in the organic phase concentration makes isocratic elution difficult because the organic phase concentration must be maintained very precisely (18-19). Furthermore, Small peptides like cetrorelix (MW: 1431) appear to chromatograph by a hybrid of partitioning and adsorption mechanisms and it make the chromatographic process more complicated (16). For this reasons, gradient elution of small peptides like cetrorelix, even with shallow gradients, is preferred, since it results in much sharper peaks than isocratic elution and isocratic elution is rarely used for peptide separations (16-20). Grohganz *et al. *reported an isocratic HPLC method for determination of cetrorelix in liposome formulation whit UV detection (9, 1 and 11). In this method cetrorelix was separated from excipients during sample preparation and due to the use of isocratic system cetrorelix was detected in short retention time (RT: 3.5 min) (9, 1 and 11). Because of the risk of interference between cetrorelix and excipients in very short run time, this method can not be used for assay of cetrorelix in its injection formulation. 

In addition, to the best of our knowledge no official report for the determination of cetrorelix in bulk material and parenteral formulation has ever been published in any pharmacopoeia. Thus, it would be of particular interest to develop and validate a simple, precise, specific, accurate, and stable gradient RP-HPLC method with UV-detector according to ICH guidelines for the determination and routine analysis of cetrorelix in pure and pharmaceutical formulations (21). 

## Experimental

*Chemicals*

Cetrorelix for injection (Cetrotide®) were prepared from Merck serono, Canada. Each vial of Cetrotide® contains 0.25 mg of cetrorelix as cetrorelix acetate and 54.80 mg of mannitol. The cetrorelix acetate was purchased from Henan New-Sensation Chemical Co., Ltd. ((Mainland, China). HPLC grade acetonitrile and TFA were from Merck (Hohenbrunn, Germany). The other reagents were of analytical grade.

*Standard preparation*

Standard stock solutions of cetrorelix acetate (1.0 mg/mL) was prepared by direct weighing of standard substance with subsequent dissolution in deionize water. The standards for the calibration curve were prepared in volumetric flasks (5mL) using standard stock solutions by serial dilution to yield concentrations of 1250, 500, 250, 125 and 62.5 μg/mL (cetrorelix as acetate).

*Quality control preparation*

A second weighing of cetrorelix acetate independent of the stock was used for the preparation of the quality control samples (QCs). This QC stock solution (1 mg/mL) was prepared in deionized water. QC samples were prepared in volumetric flasks (5ml) using QC stock solutions by serial dilution to yield concentrations of 500, 250 and 125 μg/ml (cetrorelix as acetate).

*Assay preparation*

Vials of cetrorelix for Injection (Cetrotide® 0.25 mg) were separately dissolved in 1 mL water for injection to obtain a concentration of 250 μg/mL. The drug concentration of the resulting sample solution was determined by HPLC using the calibration curve of standard solution. All determinations were conducted in triplicate. 

*Chromatographic conditions*

The liquid chromatographic system consisted of an Agilent 1200 Series HPLC System equipped with autosampler (G1329A), UV detector (G1314B), degasser (G1379B), and binary pump (G1312A) (GenTech Scientific, NY,USA). The HPLC parameters were controlled by ChemStation Software Rev.B.03.01. Analysis was carried out at 275 nm with a Lichrospher® C18, 250 × 4.60 mm, 5μm column using gradient elution of two solutions, A (0.1% (v/v) TFA in water) and B (0.1% (v/v TFA in acetonitrile). Both mobile phases were degassed by vacuum for 15 min. The following gradient program was applied: 90% A for 5min, from 90% A to 70% B in 15 min, 70% B for 10 min, from 70% B to 90% A in 5 min and 90 % A for 30 min. The flow rate was 1 mL/min, and total run time was 65 min. The injection volume was 20 μL.

*Method validation*

*Specificity*

The selectivity of the methods was evaluated by comparing the chromatograms of blank samples (deionized water and aqueous solution of mannitol, 54.8 mg/mL) versus standard samples.

*Linearity*

Six calibration standards (1250–62.5 μg/mL) were prepared covering the expected range. The standards were prepared in deionize water. The data for peak area versus drug concentration were treated by linear regression analysis.

*Sensitivity*

The sensitivity of the analytical technique was expressed as the LOQ, which is the minimum concentration of cetrorelix that can be quantitatively determined with the peak-to-noise ratio at least 10:1, and the LOD as a peak-to-noise ratio at 3:1. The LOQ is accepted if the analyte peak response is identifiable, discrete and reproducible with a precision of 2% and accuracy of 95–115% (21, 22).

*Precision and accuracy*

The intra-day and inter-day precision were determined by analyzing three replicates of quality control samples at concentrations of 125, 250 and 500 μg/mL (cetrorelix as acetate) on the same day and three times on three days, respectively. The precision was evaluated by the relative standard deviation (RSD) and the acceptable range of RSD was no more than 2% (21, 22). The accuracy was assessed by the methodological recovery. The recovery of the method was calculated by comparing the determined concentration of QC samples to the theoretical concentrations.

*Stability*

Stability of the QC samples were evaluated after short-term storage (at 25 °C for 24 h), after long-term storage (at – 20 °C for 1 week) and after going through freeze-and-thaw cycles (from −20 ^o^C to room temperature for every 24 h). The stability of standard stock solution was also evaluated.

*Statistical analysis*

The data were submitted to statistical analysis using Excel® software.

## Results and Discussion

*HPLC method development*

*Choice of detection wavelength*

**Figure 2:**
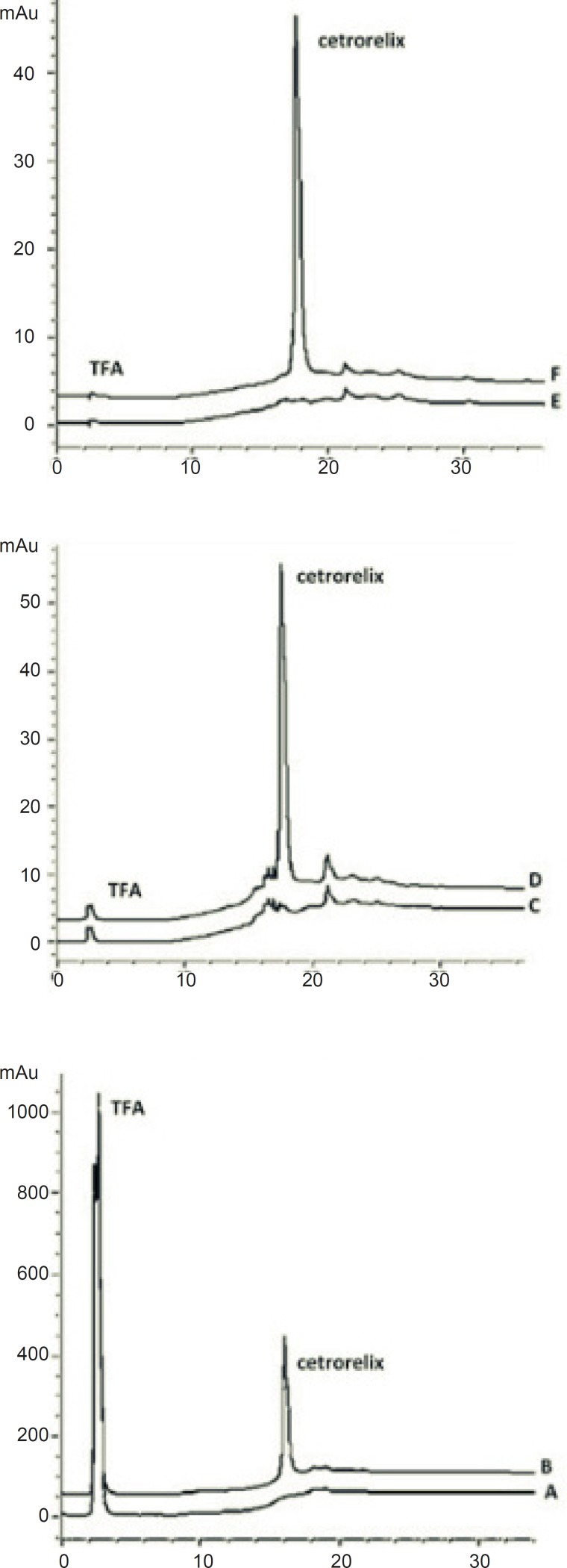
Chromatograms of: (A) blank sample (deionized water) in 230 nm (B) standard solution, 125 μg/mL cetrorelix as acetate in 230 nm (C) blank sample (deionized water) in 265 nm (D) standard solution, 125 μg/mL cetrorelix as acetate in 265 nm (E) blank sample (deionized water) in 275 nm (F) standard solution, 125 μg/mL cetrorelix as acetate in 230 nm

**Figure 3:**
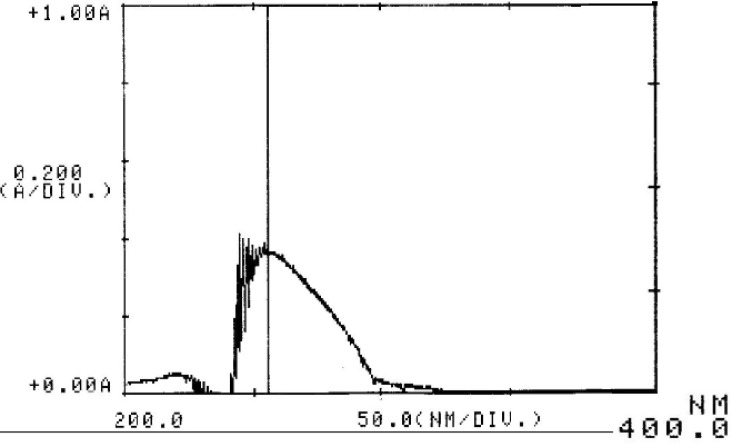
UV spectrum of cetrorelix acetate

Cetrorelix peak was monitored at different wavelengths (230, 265 and 275 nm) and a compromise wavelength of 275 nm was selected as the optimum wavelength for HPLC analysis because it maximizes the peak symmetry of cetrorelix, while giving a flat baseline and minimum signal of TFA (Figure 2). Furthermore the ultraviolet spectra of aqueous solution of cetrorelix showed the maximum absorption wavelength at 275 nm (Figure 3).

*Choice of gradient program for mobile phase*

Separation of small molecules involves continuous partitioning of the molecules between the mobile phase and the hydrophobic stationary phase. Peptides, however, are too large to partition into the hydrophobic phase; they adsorb to the hydrophobic surface after entering the column and remain adsorbed until the concentration of organic phase reaches the critical concentration necessary to cause desorption (18-19). Because peptides diffuse slowly, RP-HPLC results in broader peaks than obtained with small molecules (16). In addition, Small peptides have a hybrid separation mechanism. They desorb more quickly with changes in organic modifier concentration than small molecules which partition, however they desorb more gradually than proteins. For this reasons, gradient elution is generally preferred for small peptide separations (16, 19, 20 and 23). 

**Figure 4:**
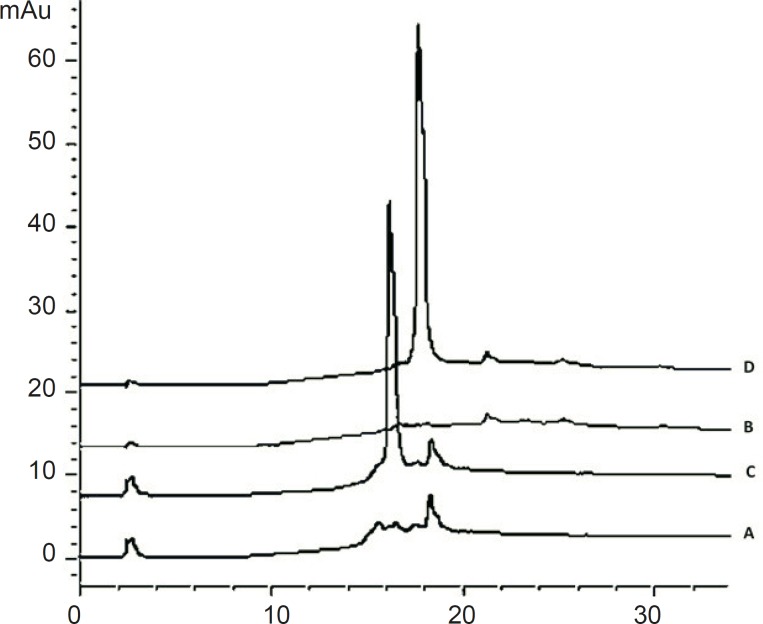
Chromatograms of: (A) blank sample (deionized water) and (B) standard solution, 125 μg/mL cetrorelix as acetate (RT: 15.9 min) with gradient of 90% A for 5min, from 90% A to 70% B in 10 min, 70% B for 10 min – (C) blank sample (deionized water) and (D) standard solution, 125 μg/ mL cetrorelix as acetate (RT: 17.5 min) with gradient of 90% A for 5min, from 90% A to 70% B in 15 min, 70% B for 10 min

Cetrorelix is a small peptide (MW: 1431) with hybrid separation mechanism. The sensitivity of cetrorelix elution to the change in acetonitrile concentration per unit time is illustrated in Figure 4. Large changes occur in the retention time of cetrorelix and base line flatness with relatively small changes in the acetonitrile concentration per unit time. The sensitivity of cetrorelix retention to subtle changes in the acetonitrile concentration makes isocratic elution difficult because the organic modifier concentration must be maintained very precisely.

**Figure 5:**
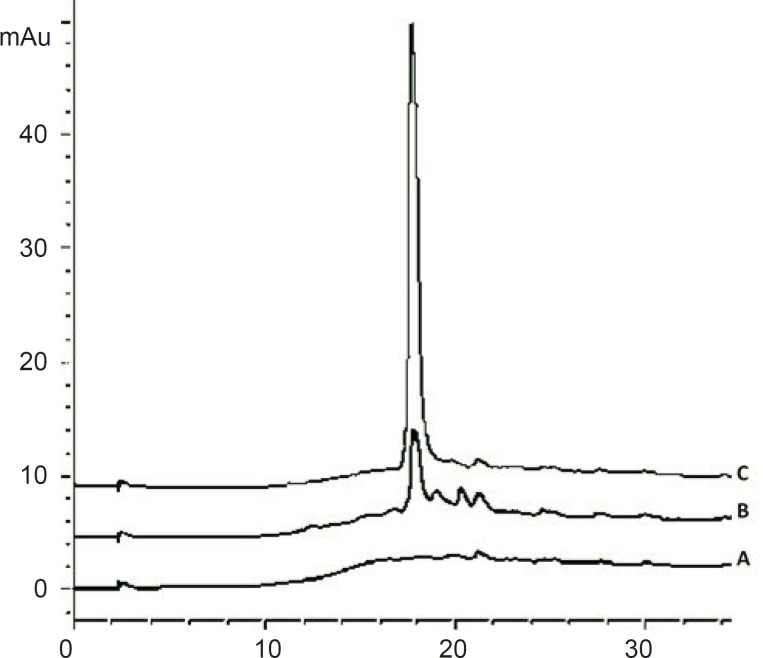
Chromatograms of: (A) blank sample (deionized water) (B) standard solution, 125 μg/mL cetrorelix as acetate in mobile phase with 0.05% TFA (C) standard solution, 125 μg/ mL cetrorelix as acetate in mobile phase with 0.1% TFA

TFA sets the eluent pH and interacts with the peptide to enhance the separation. It is normally used at concentrations of about 0.1% (v/v). TFA concentrations up to 0.5% have been useful in solubilizing larger or more hydrophobic proteins and lower concentrations are occasionally used for tryptic digest separations (24). Using TFA concentration lower than 0.1 % in mobile phase will disturb the peak shape for cetrorelix (Figure 5). 

In the current study in addition to the percentages of acetonitrile and concentration of the TFA, effects of re-equilibration time, gradient delay time and ramp time on the retention time and symmetry of peak were investigated. One factor at the time was varied while the others retained at a constant value. 

*Validation of the method*

*Specificity*

**Figure 6:**
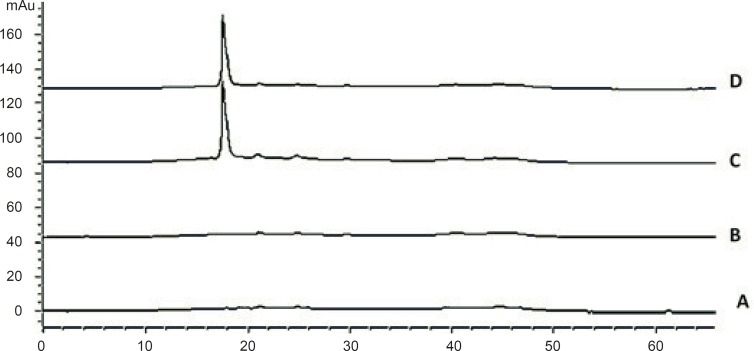
Chromatograms of: (A) blank sample (deionized water) (B) blank sample (queous solution of mannitol, 54.8 mg/mL) (C) standard solution, 250 μg/mL cetrorelix as acetate (D) Assay sample solution (Cetrotide^® ^0.25 mg in 1 mL deionized water)

The peak purity was ensured by comparing the chromatogram of the cetrorelix standard samples with that of blank samples. The HPLC chromatograms were recorded for standard samples (aqueous cetrorelix solution) and assay samples (aqueous cetrorelix solution with mannitol) and their comparison revealed no peaks in the vicinity of cetrorelix retention time (around 17.7 minutes). No signs of interference with mannitol were detected in cetrorelix for injection formulation (Figure 6). 

*Linearity*

**Figure 7:**
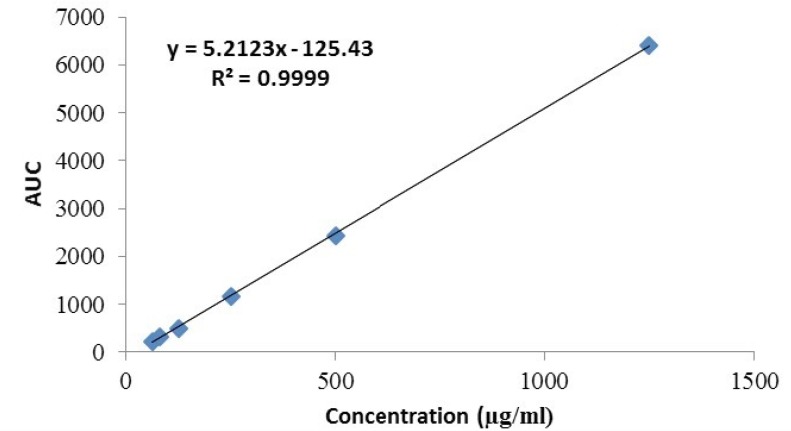
Linearity plot for cetrorelix drug substance

The linearity of the HPLC method used for cetrorelix assay was evaluated by injecting standard concentrations of cetrorelix drug substance. The plot was linear over the concentration range of 62.5 -1250 μg/mL yielding a regression equation Y= 5.2 X – 125.4 with a coefficient of correlation of 0.999 and with confidence intervals at p = 0.05 (Figure 7). 

*Limit of quantitation and detection*

The LOQ was found to be 62.5 μg/mL with signal to noise ratio of about 10. The recovery of quantitation level was 110% with an RSD less than 1.4 %, which met the validation criteria for recovery and precision. 

*Precision*

The results obtained for inter- and intraday precisions are presented in Table 1. Method precision has a RSD below 0.27% for Intraday and 1.60 % for interday precision, which comply with the acceptance criteria proposed (RSD < 2%) (21 and 22). 

*Accuracy*

Accuracy was determined by evaluating the recovery of analyte. The percent recovery between theoretical (Ctheo) and calculated (Ccalc) concentration was derived by the following equation:

Recovery (%) = Ccalc/Ctheo× 100

The accuracy of the QC samples ranged from 97 to 99%, indicating excellent accuracy of the proposed HPLC method (Table 1).

**Table 1:** Intra- and interday precision and accuracy of cetrorelix QC samples using the described HPLC method.

**Theoretical concentration**	**Calculated concentration** **(mean±S.D., n=3)**	**Precision** **(R.S.D.) (%)**	**Accuracy** **Recovery %**
**Intraday**			
125	121.6 ± 0.11	0.11	96.85
250	242.56 ± 0.40	0.17	97.03
500	485.13 ± 1.28	0.26	97.03
**Interday**			
125	121.28 ± 1.90	1.56	97.02
250	243.46 ± 2.97	1.21	97.38
500	488.56 ± 5.57	1.14	98.69

*Stability*

*Short-term stability: *QC samples were kept at room temperature for 24 h and analyzed. The accuracy for samples ranged from 95 to 98 % after short term stability testing.

*Long-term stability: *QC samples were kept at – 20 ^o^C for one week. These samples were thawed at room temperature and analyzed. The accuracy for samples ranged from 95 to 99 % after long term stability testing.

*Freeze and thaw cycles: *QC samples were prepared and frozen at −20 ◦C for 24 h. The samples were thawed at room temperature and analyzed. The samples were refrozen for 24 h under the same conditions. The freeze–thaw cycle was repeated two more times, and then analyzed on the third cycle. The accuracy for samples ranged from 94 to 99% after freeze thaw stability testing.

*Standard stock solution stability: *The standard stock solutions were found to be stable for one week when refrigerated at +4 ^o^C. The concentration on comparison with freshly prepared standard after the storage was 98.70%.

*Application of the method for the analysis of cetrorelix for injection formulation (Cetrotide* ^®^ *0.25 mg)*

The method was applied to assay cetrorelix in 10 samples of cetrorelix for injection formulation (Cetrotide^®^ 0.25 mg). Peak areas of cetrorelix were measured and amount of drug was calculated from the respective calibration plots. The average assays (n = 10) was 101.52 ± 2.62.

## Conclusion

The developed RP-HPLC method for determination of cetrorelix is simple, precise, accurate, linear, and sensitive. This method was validated based on ICH guideline (21).The measured signal was shown to be precise, accurate and linear over the concentration range tested (62.5–1250 μg/mL) with a correlation coefficient better than 0.999. The developed method showed no interference with the formulation excipient. Therefore, this method can be used for the routine determination of cetrorelix in pure and pharmaceutical formulations.
